# Sea Anemone Membrane Attack Complex/Perforin Superfamily Demonstrates an Evolutionary Transitional State between Venomous and Developmental Functions

**DOI:** 10.1093/molbev/msae082

**Published:** 2024-04-27

**Authors:** Joachim M Surm, Morani Landau, Yaara Y Columbus-Shenkar, Yehu Moran

**Affiliations:** Department of Ecology, Evolution and Behavior, Alexander Silberman Institute of Life Sciences, The Hebrew University of Jerusalem, 9190401 Jerusalem, Israel; Department of Ecology, Evolution and Behavior, Alexander Silberman Institute of Life Sciences, The Hebrew University of Jerusalem, 9190401 Jerusalem, Israel; Department of Ecology, Evolution and Behavior, Alexander Silberman Institute of Life Sciences, The Hebrew University of Jerusalem, 9190401 Jerusalem, Israel; Department of Ecology, Evolution and Behavior, Alexander Silberman Institute of Life Sciences, The Hebrew University of Jerusalem, 9190401 Jerusalem, Israel

**Keywords:** gene duplication, venom, Cnidaria, reverse recruitment, subfunctionalization, toxin

## Abstract

Gene duplication is a major force driving evolutionary innovation. A classic example is generating new animal toxins via duplication of physiological protein-encoding genes and recruitment into venom. While this process drives the innovation of many animal venoms, reverse recruitment of toxins into nonvenomous cells remains unresolved. Using comparative genomics, we find members of the Membrane Attack Complex and Perforin Family (MAC) have been recruited into venom-injecting cells (cnidocytes), in soft and stony corals and sea anemones, suggesting that the ancestral MAC was a cnidocyte expressed toxin. Further investigation into the model sea anemone *Nematostella vectensis* reveals that three members have undergone *Nematostella*-specific duplications leading to their reverse recruitment into endomesodermal cells. Furthermore, simultaneous knockdown of all three endomesodermally expressed MACs leads to mis-development, supporting that these paralogs have nonvenomous function. By resolving the evolutionary history and function of MACs in *Nematostella*, we provide the first proof for reverse recruitment from venom to organismal development.

## Introduction

In recent years, the starlet sea anemone *Nematostella vectensis* has been developed into a model system in the study of evolutionary developmental biology (Hand and Uhlinger 1992). This is due to *Nematostella* being a member of the ancient phylum Cnidaria (sea anemones, jellyfish, corals, and hydroids) that diverged from Bilateria more than 600 million years ago, yet sharing a striking level of conservation in genomic features to bilaterians, such as synteny and gene content (Cartwright et al. 2007; Putnam et al. 2007; Technau et al. 2015; Rentzsch and Technau 2016; Zimmermann et al. 2023). In addition, *Nematostella* has been established as a model to unravel the evolution and function of genes due to its ability to complete a full life cycle in the lab, its fully sequenced chromosomal-level genome, and the development of new tools for its genetic manipulation, such as transgenesis methods, gene knockdowns, and knockouts (Layden et al. 2016; Al-Shaer et al. 2021). While this has been essential in unraveling characteristics of the cnidarian–bilaterian common ancestor, it has also revealed key insights into the evolution of novel innovations, in particular the evolution of venom (Moran et al. 2015; Surm and Moran 2021; Babonis et al. 2022; McCulloch et al. 2023).

Members of Cnidaria are venomous and employ specialized organelles called nematocysts as miniature venom injectors (Kass-Simon and Scappaticci 2002). These highly complex intracellular biological structures are composed of various structural polymers that are packed in stinging cells called nematocytes; then, upon specific signals, the nematocysts discharge at remarkable speed and puncture their target (Kass-Simon and Scappaticci 2002; David et al. 2008). Among the list of cnidarian model systems, which is continuously expanding, *Nematostella*'s venom system is arguably the most well-studied (reviewed in Surm and Moran 2021). These works have revealed key insights into the molecular mechanisms underlying the nematocyte developmental origin (Steger et al. 2022; Tournière et al. 2022), such as nematocytes originating from a common neurosecretory progenitor, to their diversity, such as connecting single transcription factor (NvSox2) which can switch between two alternative stinging cell fates (Babonis et al. 2023). Genetic manipulation techniques have also been crucial in characterizing toxin genes and their novel cell types (Columbus-Shenkar et al. 2018; Sunagar et al. 2018; Sachkova et al. 2019) as well as resolving long-standing evolutionary theories related to venom biology such as resolving their impact on fitness (Surm et al. 2024).

In addition, these genetic tools have also been valuable in tracking the genesis of toxins from genes with nonvenom functions, for example, the recruitment of conserved sea anemone neuropeptide into nematocyte following a *Nematostella*-specific gene duplication event (Sachkova et al. 2020a). This process is known as “recruitment,” and it is assumed that many toxins originate from gene duplication of proteins that carry physiological roles, with their new paralogs undergoing neofunctionalization to become a toxin (Fry et al. 2009). Originally, recruitment into venom was described as a one-way process, where physiological genes transform into toxins; however, a previous study in squamate reptiles hinted that this may indeed be a two-way process where “reverse recruitment,” i.e. the transformation of a venom protein back into a physiological nonvenom protein, can also occur (Casewell et al. 2012). However, reverse recruitment has yet to be proven experimentally.

In this work, we studied members of the membrane attack complex and perforin family (MACPF) in *Nematostella* (we refer to these as MAC). This family includes β-pore-forming toxins (PFTs) that are found in a wide variety of organisms from bacteria to mammals and are mostly employed for lysing cells by generating pores in their membranes (Anderluh et al. 2014). MACs were found to be toxins in the nematocysts of two sea anemones, *Actineria villosa* and *Phyllodiscus semoni* (Nagai et al. 2002; Oshiro et al. 2004; Satoh et al. 2007; Uechi et al. 2011). Using comparative genomics and phylogenetics, as well as interrogating the publicly available cell atlases, we find that members of MAC were recruited into the nematocytes of the last common ancestor of Anthozoa (soft corals, stony corals, and sea anemones). We find that following two rounds of lineage-specific duplications in *Nematostella*, three additional MAC paralogs were recruited into endomesodermal cells strongly suggesting that they carry nonvenomous functions. This is further supported by evidence that depleting these three endomesodermal MACs interferes with normal development in *Nematostella*. Additionally, two of these endomesodermal MAC paralogs still retain some weak expression in stinging cells and represent a “transitional form” between a toxin and a nonvenom protein-encoding gene. These findings are the first experimental proof of the reverse recruitment of venom and highlight the power of gene duplication in the rapid evolution of molecular innovation.

## Results

### Evolutionary History of the MAC Family across Anthozoa

**Fig. 1.**
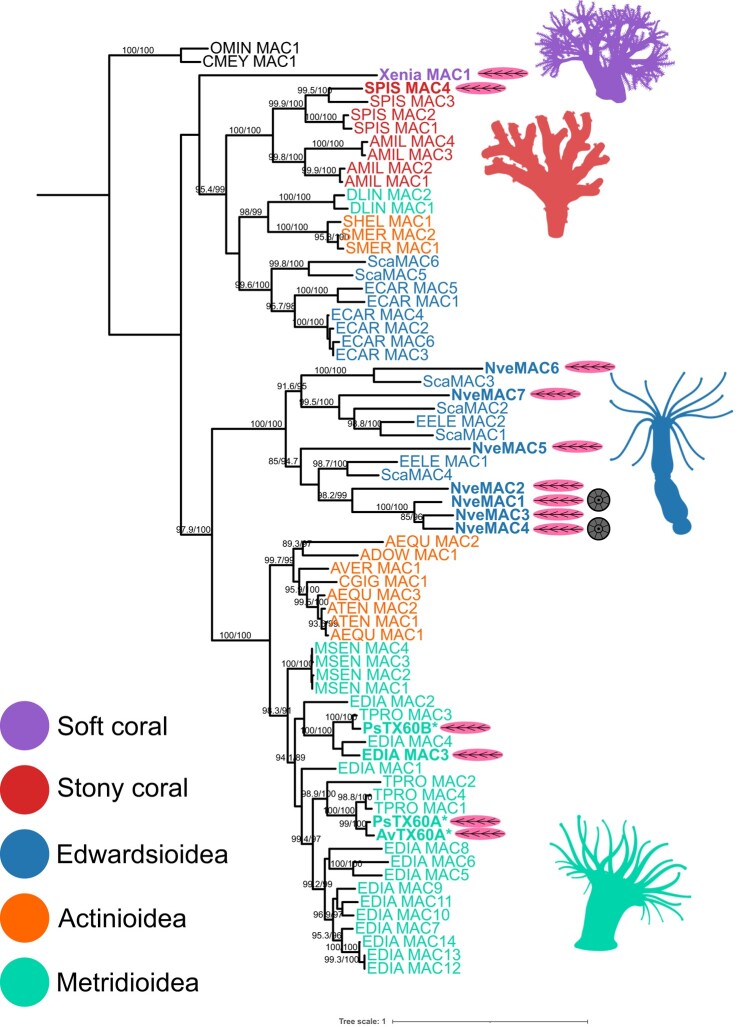
Phylogeny of MAC genes across Anthozoa. Maximum-likelihood tree of the MACs in anthozoans. The sequences found in nematocytes appear in bold with a magenta/elliptical nematocyte cartoon. The nematocyte-specific expression is characterized using cell atlases from different anthozoans including *Xenia* sp., *S. pistillata*, *E. diaphana*, and *Nematostella* (Hu et al. 2020; Levy et al. 2021; Steger et al. 2022; Cui et al. 2023). Additional NveMACs are found to have nematocyte-specific expression using bulk RNA-seq of the *Nematostella* transgenic line expressing NvNcol3::memOrange2, a nematocyte marker (Sunagar et al. 2018; Gahan et al. 2022). Sequences found to have high levels of expression in the Endo-atlas of *Nematostella* (He et al. 2023) also contain a gray/circular cartoon representing the endomesodermal segments. MACs found in the venom and isolated from nematocytes of anthozoans are highlighted with an asterisk as well as in bold with a magenta nematocyte cartoon. Ultrafast bootstrap values and SH-like approximate likelihood ratio test above 85 are indicated. Taxon identifies can be found supplementary table S1, Supplementary Material online. Silhouettes were made from BioRender.com.

Using a phylogenetic framework, we investigated the distribution of MAC genes across Anthozoa. To do this, we used a combination of predicted proteomes generated from anthozoans with sequenced genomes, as well as additional transcriptomes from sea anemones (supplementary table S1, Supplementary Material online). Specifically, we find in the *Nematostella* (from the Edwardsioidea superfamily) genome seven sequences that encode for proteins composed of a signal peptide and a single MACPF domain, which we named NveMAC1 through 7 (Fig. 1). Similarly, we find that the MAC gene family has undergone numerous amplification events in other Hexacorallia genomes, resulting in the presence of four copies in the stony corals *Stylophora pistillata* and *Acropora millepora* each. Among sea anemone genomes, we find considerable variation in their copy number, with six copies in the other edwardsioidean: *Scolanthus callimorphus*, 14 copies in metridioidean: *Exaiptasia diaphana*, and two and three copies in the actinioideans: *Actinia tenebrosa* and *Actinia equina*, respectively.

Next, we generated a maximum-likelihood tree which reveals the presence of two distinct clades, one clade including only sea anemones and the other including soft corals, stony coral, and sea anemones (Fig. 1). Within both clades, the broad clustering is consistent with anthozoan phylogeny, with Octocorallia (soft corals) being the most diverged compared with Hexacorallia (which includes stony corals and sea anemones). Within the sea anemone-specific clade, the superfamilies Metridioidea and Actinioidea cluster together and species from Edwardsioidea are the most diverged. This topology is also consistent with actiniarian phylogeny. Beyond this broad clustering, we see repeated evidence of species-specific clustering, in Hexacorallia, suggesting lineage-specific duplications are underlying much of the evolution of this gene family. For example, we find that *Nematostella* (Nve) MACs 1 to 4 cluster together, in *E. diaphana* MACs 5 to 14 cluster together, and all *S. pistillata* and *A. millepora* sequences cluster in a species-specific manner. We also find evidence of gene loss events which is likely contributing to the patchy distribution of sea anemone sequences in the anthozoan clade which includes sequences from the genome of *S. callimorphus* but also sequences from the transcriptomes of species coming from all three actiniarian superfamilies. This suggests that gene loss events happened independently in both *Nematostella* and *E. diaphana*, suggesting that the evolution of the MAC gene family is highly dynamic.

**Fig. 2.**
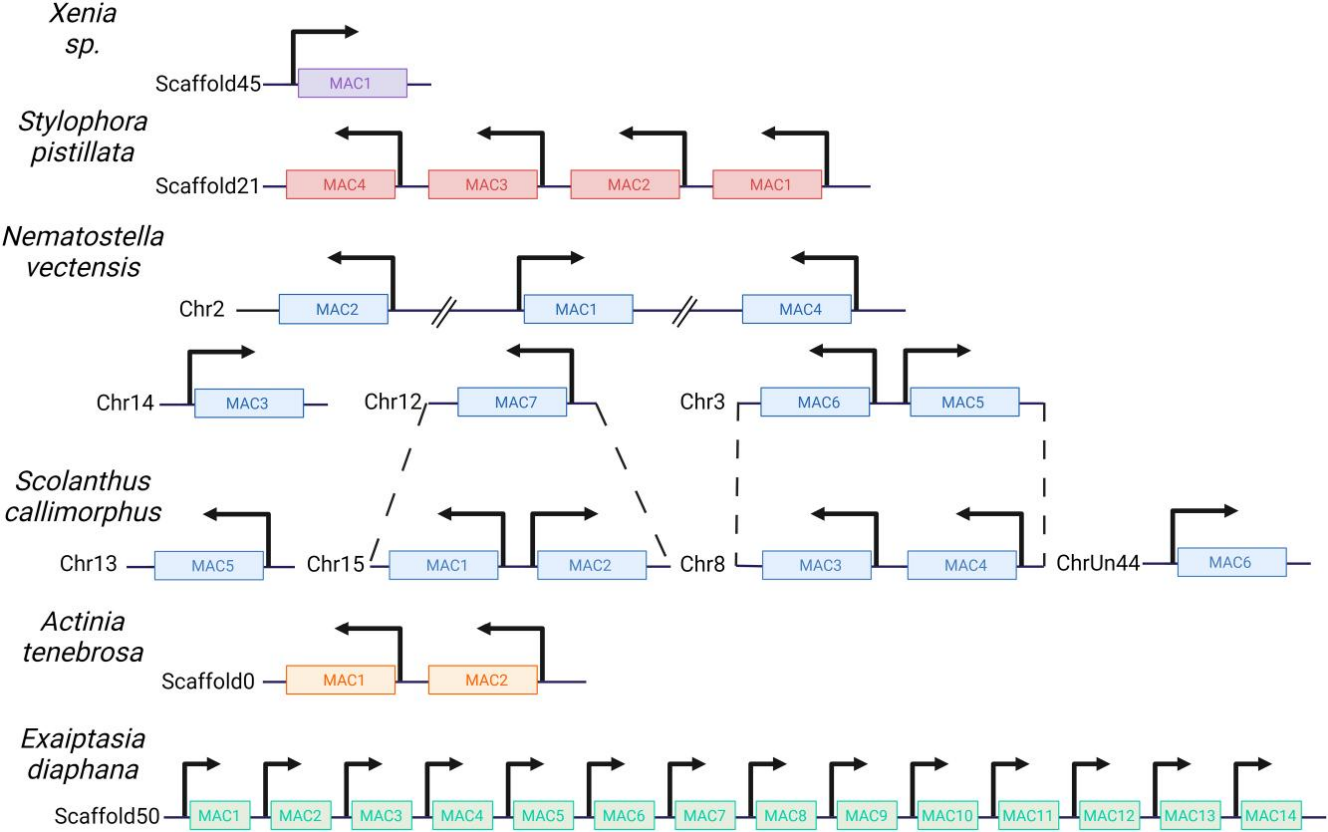
Schematic representation depicting the MAC gene cluster and gene synteny among Anthozoans. Dashed lines between *N. vectensis* and *S. callimorphus* represent a cluster that shares macrosynteny. Created with BioRender.com

While we report lineage-specific duplications among *Nematostella* MACs 1 to 4, we also find that some *Nematostella* and *S. callimorphus* MACs are orthologous, cluster together phylogenetically, as well as share chromosomal macrosynteny (Fig. 2, supplementary table S2, Supplementary Material online). Specifically, we find that NveMACs 5 and 6 are orthologous with *S. callimorphus* (Sca) MACs 4 and 3, respectively. They also share macrosynteny, with NveMAC5 and 6 being found on chromosome 3 in *Nematostella* which is homologous to chromosome 8 in *S. callimorphus* where ScaMAC 3 and 4 are found. We also find that NveMAC7 is orthologous with ScaMAC1 and ScaMAC2, clustering together as well as sharing macrosynteny (Fig. 1). This strongly supports that NveMACs 5 to 7 are likely the ancestral sequences, with MACs 1 to 4 evolving via *Nematostella*-specific duplications.

MACs from the three sea anemone superfamilies investigated can be found in both MAC clades. The clade containing only sea anemone sequences, however, includes proteins known to be a component of sea anemone venom profiles. Specifically, the sequences from *P. semoni* (PsTX60A and PsTX60B) and *A. villosa* (AvTX60B) have been isolated from nematocytes and shown to be toxic to shrimp and hemolytic toward sheep red blood cells (Nagai et al. 2002; Oshiro et al. 2004; Satoh et al. 2007; Uechi et al. 2011). Using AlphaFold, analysis of the predicted structure of NveMACs 1 to 4 with the predicted structure of PsTX60A and PsTX60B showed broad overlap among all sequences, suggesting that potentially NveMACs 1 to 4 might also have hemolytic activity (supplementary fig. S1, Supplementary Material online). This hinted to us that these sequences may therefore also play a role in the venom composition of other sea anemones including *Nematostella*.

### Spatiotemporal Expression of MAC in *Nematostella*

The recent advancements in single-cell sequencing in nonmodel organisms have allowed the establishment of cell atlases of multiple different anthozoans including *Xenia* sp., *S. pistillata*, *E. diaphana*, and *Nematostella* (Hu et al. 2020; Levy et al. 2021; Steger et al. 2022; Cui et al. 2023). Strikingly, we find at least one MAC is expressed in nematocytes from each of the anthozoan cell atlases (Fig. 1, supplementary table S1, Supplementary Material online). In *Nematostella*, which arguably has the most comprehensive cell atlas, generated using a detailed scRNA-seq data set across development, we find that NveMACs 1 to 3 have expression in nematocytes. To complement this, we investigated bulk RNA-seq of the *Nematostella* transgenic line expressing *NvNcol3::memOrange2*, a nematocyte marker (Sunagar et al. 2018; Gahan et al. 2022). We find that all *Nematostella* MACs are upregulated in NvNcol3::memOrange2 positive cells, further supporting that *Nematostella* MACs are expressed in nematocytes, including the more ancestral sequence NveMACs 5, 6, and 7.

Finally, a recent endomesodermal-enriched scRNA-seq data set from planula was generated to construct a 3D spatial gene expression atlas of *Nematostella*. From this data set, we find that two MACs, MACs 1 and 4, are expressed at relatively high levels (>10) compared with the other NveMACs, as well as all other *Nematostella* toxins, which all had low to no expression in the Endo-atlas (supplementary tables S1 and S3, Supplementary Material online). This finding suggests that these two NveMACs are expressed in the endomesoderm in addition to nematocytes. This is further supported by RNA-seq data from previous experiments where loss-of-function mutations in HOX genes (ANthox1a, ANthox6a, ANthox8), which are important regulators of endomesodermal segmentation, were introduced to *Nematostella* embryos (He et al. 2023). The sequencing revealed that those mutations resulted in significant down regulation of NveMAC1 and NveMAC4 (supplementary table S8, Supplementary Material online). No other NveMACs or *Nematostella* toxins were found to be dysregulated following the knockout of the HOX genes. However, a similar pattern is observed for other genes known to be expressed in the endomesoderm (He et al. 2023), further supporting that NveMAC1 and NveMAC4 are expressed in the endomesoderm.

**Fig. 3.**
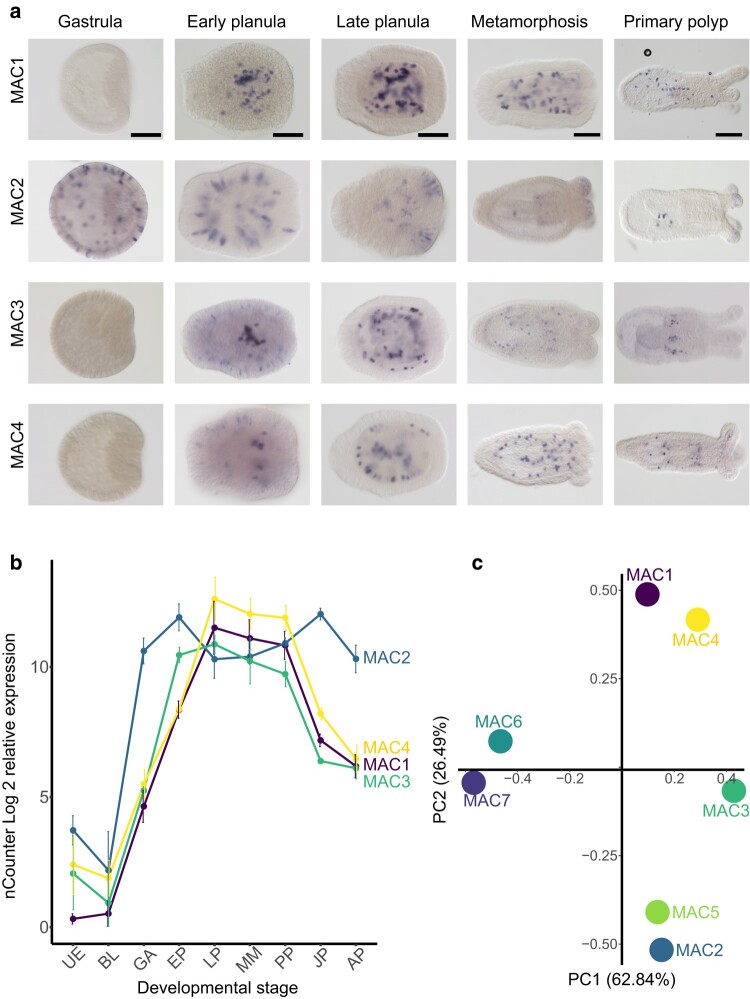
Spatiotemporal expression of MAC-encoding genes in *Nematostella*. a) ISH of NveMACs (1 to 4) across embryonic development. b) Graph of RNA levels of the same MAC genes used in ISH. Scale bar represents 100 μm. c) PCA using temporal expression of NveMACs in *Nematostella* throughout embryonic development taken from the NvERTx database. UE, unfertilized egg; BL, blastula; GA, gastrula; EP, early planula; LP, late planula; MM, metamorphosis; PP, primary polyp; JP, juvenile polyp; AP, adult polyp.

Due to the conflicting results coming from different *Nematostella* single-cell and RNA-seq data sets which revealed that NveMAC1 and 4 are likely expressed in both endomesodermal cells and nematocytes, we aimed to characterize the expression of NveMACs experimentally using in situ hybridization (ISH). Interestingly, of the four genes that are expressed at levels detectable by ISH, only NveMAC2 is being expressed exclusively in nematocytes, whereas we find that NveMAC1 is expressed exclusively in endomesodermal cells (Fig. 3a). To verify the specificity of the obtained ISH patterns, we microinjected to *Nematostella* zygotes short-hairpin RNA (shRNA) against NveMAC1 and showed that the staining is greatly reduced in comparison with larvae of the same age that developed from zygotes injected with control shRNA (supplementary fig. S2, Supplementary Material online): NveMAC1 knockdown: 3/189 stained embryos; control shRNA, 145/146 stained embryos. In parallel, we explored the temporal expression of NveMACs using NanoString nCounter data, revealing that NveMAC1 expression is restricted to a very short period, peaking at the planula stage (Fig. 3b and supplementary fig. S3 and tables S4 and S5, Supplementary Material online). In contrast, NveMAC2 expression is relatively stable throughout the life of *Nematostella*. To support this, we extracted RNA-seq count data across early development (0 to 240 hpf) from the NvERTx database. (Helm et al. 2013; Fischer et al. 2014; Warner et al. 2018) and performed principal component analysis (PCA) to help quantify and visualize the temporal expression of NveMACs. The PCA confirms that NveMAC1 and NveMAC2 have distinct temporal expression patterns evident with each grouping separately (Fig. 3c). Taken altogether, NveMAC1 and NveMAC2 have distinct spatiotemporal expression patterns, with NveMAC2 likely playing a role in *Nematostella* venom evident by its expression in nematocytes and stable expression across the life history of *Nematostella*, whereas the relatively short time window of the expression of NveMAC1 and expression in endomesodermal cells raises the unexpected possibility that it might be involved in development.

Exploring the spatiotemporal expression of NveMAC3 and 4 reveals they exhibit an “intermediate” expression pattern between NveMACs 1 and 2. In the early planula, they are weakly expressed in nematocytes, similar to NveMAC2, and strongly expressed in endomesodermal cells, resembling the same cells that express NveMAC1 (Fig. 3a). Furthermore, we find that NveMAC4 temporal expression pattern mirrors the expression pattern of NveMAC1, evident by its grouping with NveMAC1 in our PCA (Fig. 3c). Interestingly, NveMAC3 temporal expression groups with NveMACs 1, 2, and 4 on PC1 axis but uniquely in between NveMAC1 and 4 and NveMAC2. This further suggests that NveMAC3 temporal expression pattern is a combination of NveMAC1 and NveMAC2s. These findings regarding the spatiotemporal expression of NveMACs 3 and 4 suggest that they may also have an intermediate function, overlapping with both NveMAC2 in the nematocyte and playing a role in the venom arsenal of *Nematostella*, as well as potentially playing a role in development like NveMAC1.

### MAC Depletion Interferes with *Nematostella* Development

To investigate the functional role of NveMACs 1, 3, and 4, we depleted their expression in embryos by injecting shRNA targeting NveMACs specifically. Knockdown efficiency was confirmed in 4-day-old planula using qPCR, revealing that all shRNA used resulted in significant knockdown efficiency (supplementary table S6, Supplementary Material online; >50% knockdown and *P*-value < 0.05) of the target NveMAC compared with animals injected with control shRNA. After confirming the knockdown efficiency, additional animals were tracked until 10 d postfertilization (dpf). The control shRNA-injected embryos developed normally, undergoing metamorphosis and progressing into primary polyps. shRNA-injected embryos targeting specific NveMACs (1, 3, and 4) also resulted in normal developments, with animals undergoing normal metamorphosis (supplementary table S7, Supplementary Material online).

**Fig. 4.**
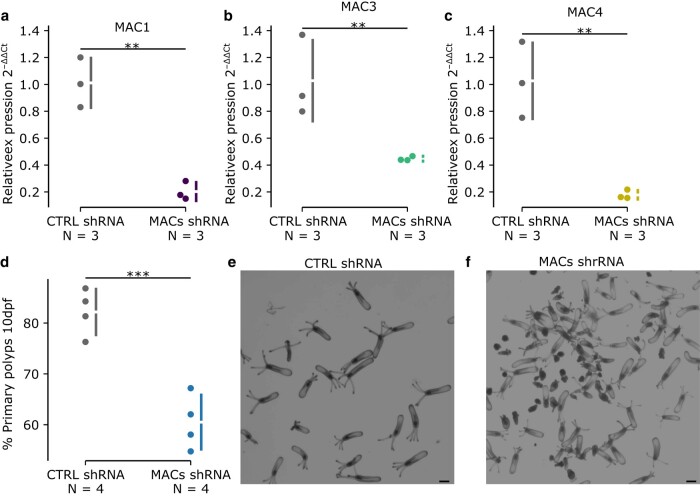
Knockdown of MACs in *Nematostella*. qRT-PCR for NveMACs at late planula stage after shRNA injection. The graph shows the relative fold change in the expression between control shRNA and the combined knockdown of a) NveMAC1, b) NveMAC3, and c) NveMAC4, using sequence-specific shRNA. The values for the individual replicates are shown as circles. The mean difference is depicted as a dot; the 95% confidence interval is indicated by the ends of the vertical error bar. d) Quantification of normal polyp development following the injection of control shRNA or knockdown of NveMACs 1, 3, and 4 simultaneously in 10 dpf polyps. e) Control shRNA. f) NveMACs 1, 3, and 4 shRNA. *P-value < 0.05; NS, not significant; **P-value < 0.01; ***P-value < 0.001. Scale bar represents 200 μm.

Given the overlap of RNA expression for NveMACs 1, 3, and 4 in the endomesoderm, we suspected that some compensation might be occurring. To test this, we coinjected shRNAs to target NveMACs 1, 3, and 4 simultaneously. We confirmed that this approach still results in a significant knockdown (Fig. 4a to c, >50%; *P*-value < 0.05) of all three NveMACs (1, 3, and 4) compared with planula injected with an equal concentration of control shRNA. Strikingly, embryos injected with a combination of shRNAs targeting MACs 1, 3, and 4 have developmental defects, with only ∼65% of 10 dpf of these animals developing into primary polyps compared with control (Fig. 4d to f, *P*-value = 0.0008), in which 85% of animals underwent metamorphosis into primary polyps by the same time point. Developmental defects in NveMACs 1, 3, and 4-depleted animals suggest these members of the MAC family are essential for proper *Nematostella* development.

## Discussion

In this work, we unraveled the evolution of a gene family of proteins containing the MACPF domain in *Nematostella* as its role transitions from venom to development. Broadly, proteins that contain the MACPF domain are part of a superfamily of PFTs that have a large distribution, covering various groups of the tree of life, from bacteria to mammals (Anderluh et al. 2014; Moreno-Hagelsieb et al. 2017). The structure of MACs enables their function in lysing cells by generating pores in their membranes (Anderluh et al. 2014; Moreno-Hagelsieb et al. 2017). This ability to cause pores in cell membranes is useful for a variety of roles in eukaryotes ranging from immunity to development (Anderluh and Lakey 2008). Two prominent examples are Astrotactins from mammals and Torso-like from flies that carry important developmental roles (Berkowicz et al. 2017; Johnson et al. 2017). Notably, some proteins that contain a MACPF domain, such as Astrotactin-2, can carry functions even if they are unable to lyse cells (Ni et al. 2016; Moreno-Hagelsieb et al. 2017).

The MACPF domain-containing proteins have undergone considerable variation at the sequence level, yet their predicted 3D structure is relatively well conserved. Notably, the sequences from *P. semoni*, which are known to lyse blood cells, share this high structural similarity (Nagai et al. 2002; Satoh et al. 2007). Given this conservation in structure, we suspect that all seven *Nematostella* MACs maintain the capacity to lyse cells, especially copies 1 to 4 which share the greatest similarity to *P. semoni* MACs. This ability to disrupt cell membranes can serve different cellular functions, including embryonic development (Anderluh and Lakey 2008). We find that development is disrupted when MAC1, 3, and 4 are depleted simultaneously. A potential explanation for this finding is that these genes are redundant and the expression of even one of them is enough to allow normal development. The recruitment of several MACs into developmental processes is a striking example for neofunctionalization.

**Fig. 5.**
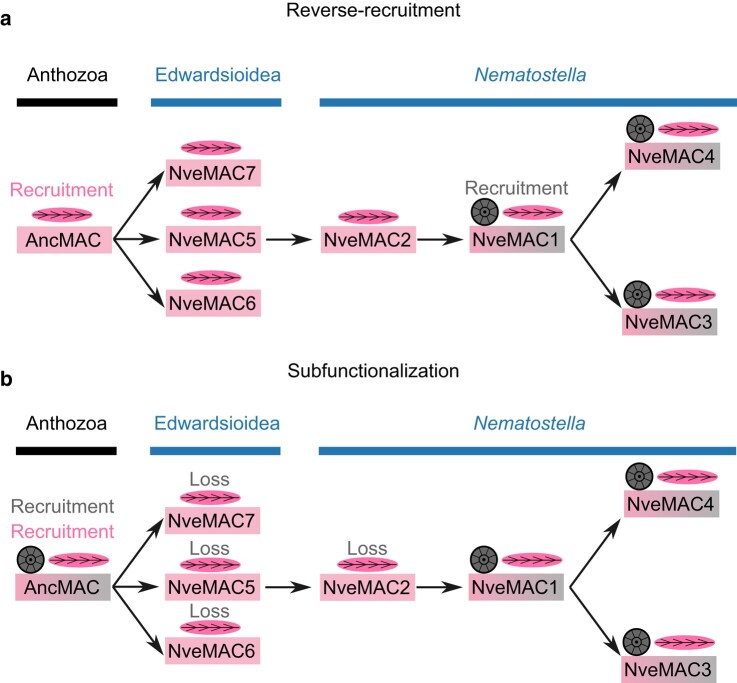
Reconstructing evolutionary history and possible scenarios of the origin of NveMACS as either reverse recruitment (a) or subfunctionalization (b). Horizontal arrows represent duplication event. Nematocyte expression is represented as magenta boxes and nematocyte cartoon, endomesodermal expression is represented by gray boxes, and cartoon represents the endomesodermal segments. Expression in both cell types is represented by boxes with both pink and gray and cartoons depicting both nematocyte and endomesodermal segments.

Reconstructing the evolutionary steps that have led generation of a gene family is a complex and challenging process, largely due to the various potential trajectories that can occur following gene duplication. For the NveMAC gene family, we have predicted two possible evolutionary scenarios which rely on different molecular processes: reverse recruitment and subfunctionalization (Fig. 5a and b). However, to disentangle between these two scenarios, we first need to reconstruct the phylogenetic history of the NveMAC copies present in the *Nematostella* genome. From our macrosynteny and phylogenetic analysis, we find the NveMACs 5 to 7 are likely the ancestral sequences due to them being orthologous to other sequences from the Edwardsioidea superfamily. Furthermore, NveMAC6 is found to neighbor NveMAC5, suggesting that NveMAC5 evolved from a tandem duplication of NveMAC6. Understanding this evolutionary history of NveMACs allows us to then reconstruct the scenarios that led to the observed function and expression domains that we find in the different NveMACs.

For the first scenario, the most parsimonious explanation is that the ancestral sequence of NveMACs 1, 3, and 4 likely was expressed in both nematocytes and endomesodermal cells. For this to occur, the ancestral MAC (AncMAC) found in the last common ancestor of all anthozoans was recruited into the nematocytes first (Fig. 5a). Multiple rounds of gene duplications eventually led to the birth of the AncMAC1/3/4 gene which was reverse-recruited out of nematocytes and venom into endomesodermal cells to function in development. Following duplication leading to the birth of MAC1 and MAC4, we suspect that the residual expression of NveMAC1 in nematocytes was nearly lost as now it is so low that it cannot be detected by ISH. This supports that MAC1 is undergoing specialization in the endomesodermal cells. Taken together with evidence that MAC1 and MAC4 share very similar temporal expression patterns, it suggests that the specialization we see in NveMAC1 occurred after the duplication and generating MACs 1 and 4. We also suspect that specialization of MAC3 and MAC4 is also occurring with the nematocyte expression being lost; however, this loss is occurring via a more gradual process.

Alternatively, subfunctionalization may also explain the evolutionary trajectory of NveMACs. Although a relatively rare event following gene duplication, subfunctionalization is a process where the gene duplicates become specialized via partitioning of the ancestral functions (Lynch and Force 2000). In the case of NveMACs, a process of subfunctionalization would hypothesize that in the last common anthozoan ancestor, the AncMAC was recruited into both endomesodermal cells and nematocytes (Fig. 5b). Having expression in both the endomesoderm and nematocytes was then maintained following repeated duplication events until NveMAC1, and NveMACs 2, 6, and 7 underwent specialization to be expressed almost exclusively in the endomesoderm and nematocytes, respectively. This would also suggest the combined expression we find in NveMACs 3 and 4 is reflecting the AncMAC expression. For this scenario to be true, it would require the ancestral expression in the endoderm and nematocyte to be retained following four duplication events until the birth of NveMAC1, with all previous copies becoming specialized to be expressed in nematocytes after the duplication event. This is plausible only if the increased expression of NveMACs in the endomesoderm is deleterious, which would cause strong selection pressure to lose the endomesodermal expression following the birth of a new NveMAC. If indeed the ancestral NveMAC had expression in both endomesoderm and nematocytes, it would also assume that orthologs of AncMAC would also have expression on both cell types. This scenario would therefore assume that a MAC found in the last common ancestor of Anthozoa had expression in both endomesoderm and nematocytes and became specialized to nematocytes independently four times. However, we found no evidence that members of this MAC gene family had expression in any other cell type. Our alternative hypothesis of reverse recruitment is more parsimonious in which AncMAC was expressed in nematocytes and gained dual expression in NveMAC1, which later specialized to just the endomesoderm, and this dual expression has also been retained in NveMAC3 and 4. Furthermore, the reverse recruitment scenario does not require any assumptions regarding deleterious effects of MAC expression in the anthozoan endomesoderm, making it more plausible.

It should be noted that while we see no evidence of nematocyte expression of NveMAC1 in our ISH results, the scRNA-seq analysis suggests that this gene still exhibits some expression in nematocytes. This residual expression is caught by ISH for NveMAC3 and NveMAC4, suggesting it is more profound for these genes. The very weak expression of NveMACs 1, 3, and 4 in cnidocytes compared with their endomesodermal expression domain suggests that this is “vestigial expression” that might becoming nonfunctional. Furthermore, the expression of NveMAC3 and NveMAC4 both in nematocytes and in endomesodermal cells suggests that they may be a molecular transitional state between the ancestral expression domain in nematocytes to the derived expression domain in the endomesoderm.

Plausibly, maintaining the ancestral nematocyte expression of NveMAC3 and NveMAC4 may not be deleterious and therefore is kept due to neutrality. Our phylogenetic analysis suggests that these genes arise from more recent duplication events in which not enough time has occurred for them to lose this ancestral nematocyte expression via drift. This is consistent with previous systematic studies investigating gene duplicates in fungi: In this work, the authors show that genes involved in complex interactions, such as those essential for cell growth, are sensitive to increased gene expression noise associated with increased copy number and tend to not evolve via gene duplication (Wapinski et al. 2007). Contrastingly, genes that are responsive to stress and have dynamic gene expression levels tend to evolve via gene duplication. This is consistent with the function of venom-related genes, which are highly responsive to stress in *Nematostella* and have undergone significant copy number variation across populations to meet their ecological requirements (Sachkova et al. 2020b; Smith et al. 2023; Surm et al. 2024).

Overall, our findings have uncovered the striking evolutionary history of this gene family containing a MACPF domain in Anthozoa. We reveal that gene duplication is driving the recruitment of different members of this gene family into different cell types ranging from nematocytes, neurons, and endomesodermal cells. We have also discovered that members that have undergone more recent duplication events are currently going through a transitional state, where they have gained expression in a new cell type while still maintaining residual expression from their ancestral copy. This transitional state is an exciting discovery as piecing together the exact evolutionary process that leads to genes deriving their function is extremely difficult. The mechanistic basis for the expression of these genes in multiple spatial domains remains to be discovered.

## Methods

### Comparative Genomics and Phylogenetics

We analyzed transcriptomes from ten sea anemone species, spanning three of the five Actiniarian superfamilies (Actinioidea, Edwardsioidea, and Metrioidea). These transcriptomes that were sampled from either multiple tissues or tentacles were downloaded from the National Center for Biotechnology Information (NCBI) Sequence Read Archive (SRA) using FASTQ-DUMP in the SRA toolkit. Raw reads retrieved were assessed for quality and trimmed using Trimmomatic (Bolger et al. 2014). Trinity was used to assemble transcriptomes de novo from the filtered raw reads (Grabherr et al. 2011; Haas et al. 2013). BUSCO (v4) was used to validate the quality and completeness of the transcriptomes (Manni et al. 2021). We predicted open-reading frames from each transcriptome using ORFfinder (https://www.ncbi.nlm.nih.gov/orffinder/) and performed BLASTp (*E*-value 1e^−05^) using sea anemones MAC toxins as queries. Sequences retained were then used to determine the presence of a signal peptide using SignalP (v5.098) as well as a single MACPF Pfam domain (PF01823.22). A similar pipeline was also performed to identify MACs across Anthozoa using genomes from *Xenia* sp., *A. millepora*, *S. pistillata*, *N. vectensis*, *S. callimorphus*, *A. tenebrosa*, *A. equina* and *E. diaphana* (CC7). Sequences were then manually curated, and those including large insertions or deletions were removed.

The refined list of full-length MACs was used for phylogenetic analyses to determine the reconstruct its evolutionary history across Anthozoa. Protein sequences were aligned using MUSCLE in MEGA 11 (Tamura et al. 2021). Protein alignments were imported into IQ-TREE, and the best fit of protein model evolution was determined using ModelFinder (Nguyen et al. 2015). Using the Bayesian information criterion, a WAG+I+G4 model was selected as the best-fit model of protein evolution. Phylogenetic trees were generated from alignments using 1,000 ultrafast bootstrap iterations and the SH-aLRT test (Guindon et al. 2010). The tree was visualized using Interactive Tree Of Life (Letunic and Bork 2016).

Homologous chromosomes were found among the genomes to determine if anthozoan MACs show any evidence of macrosynteny among anthozoans. This was achieved first by identifying 1,103 single-copy orthologs using OrthoFinder (Emms and Kelly 2019) with the predicted proteomes annotated from anthozoan genomes that were generated using long-read sequencing technology. This included *A. millepora*, *Nematostella*, *S. callimorphus*, and *A. tenebrosa*. The chromosomal locations for the single-copy orthologs were then compared to generate a macrosynteny map of chromosomes among the genomes. For the remaining genomes, we extracted all predicted proteins from the same scaffolds containing MACs and performed BLASTp (1e^−5^) against *Nematostella* proteins and counted their chromosomal location in the *Nematostella* genome. For *E. diaphana*, scaffold50 was found to contain all MACS used in this study and was downloaded from Reef Genomics (http://aiptasiav2.reefgenomics.org/). Proteins were then predicted using the FGENESH (Solovyev et al. 2006) online server (http://www.softberry.com/berry.phtml).

### Sea Anemone Culture

*Nematostella* embryos, larvae, and juveniles were grown in 16‰ sea salt water at 22 °C. Adults were grown in the same salinity but at 17 °C. Polyps were fed with *Artemia salina* nauplii three times a week. Induction of gamete spawning was performed according to a published protocol (Genikhovich and Technau 2009a). The gelatinous egg sack was removed using 3% L-cysteine (Merck Millipore, Burlington, MA) and followed by microinjection of shRNAs. All *Nematostella* individuals used in this study belonged to the common lab strain originating from Rhode River MD (Hand and Uhlinger 1992).

### nCounter Analysis

Total RNA from different developmental stages of *Nematostella* was extracted as previously described (Columbus-Shenkar et al. 2018). Briefly, RNA was extracted using Tri-Reagent (Sigma-Aldrich, St. Louis, MO) according to manufacturer's protocol, treated with TURBO DNAse (Thermo Fisher Scientific, Waltham, MA) and then re-extracted with Tri-Reagent. RNA quality was assessed on Bioanalyzer Nanochip (Agilent, Santa Clara, CA). Each sample was prepared from hundreds of specimens in order to normalize for any individual variation. Gene expression of MACs was analyzed using the nCounter platform (NanoString Technologies, Seattle, WA; performed by Agentek Ltd., Israel) as previously described (Columbus-Shenkar et al. 2018), using technical triplicates, each made from a different batch of RNA sample. For each MAC transcript tested, we used two probes each. Normalization was performed using the geometric mean of the expression levels of five reference genes with stable expression across development (Columbus-Shenkar et al. 2018).

### shRNA Generation and KD Experiments

Two shRNA precursors for each MAC gene were designed as previously described (Karabulut et al. 2019; Lewandowska et al. 2021). Reverse complement sequence of shRNA precursors were synthesized as DNA ultramer oligo by Integrated DNA Technologies (Coralville, IA), mixed with T7 promoter primer in 1:1 ratio in a final concentration of 25 µM, denatured at 98 °C for 5 min, and cooled to 24 °C. shRNAs were synthesized with AmpliScribe T7-Flash Transcription Kit (Epicentre, Charlotte, NC) for 15 h followed by 15 min treatment with 1 µL of DNase I. The in vitro transcribed products were purified using the Quick-RNA Miniprep Kit (Zymo Research, Irvine, CA). shRNAs were used for microinjection at concentrations ranging from 400 to 1,200 ng/uL. Approximately 100 injected planula (4 dpf) were flash frozen in liquid nitrogen and stored at −80 °C and used for downstream qPCR analysis. MACs1 to 4 were first targeted individually, and then, MAC1, 3, and 4 were targeted simultaneously by combining three validated shRNAs each targeting MAC1, 3, or 4 specifically.

### Reverse-Transcription Quantitative PCR

To quantify the knockdown efficiency of our shRNAs, we analyzed the expression levels of MACs using reverse-transcription quantitative PCR (RT-qPCR). A minimum of three biological was used for each shRNA or combination of shRNAs. First, RNA was extracted from injected embryos following the same protocol as previously described (Lewandowska et al. 2021). Five hundred ng of RNA was converted into cDNA in a 20 μL reaction. cDNA was constructed using iScript cDNA Synthesis Kit (Bio-Rad, Hercules, CA) according to the manufacturer's protocol. Real-time PCR was prepared with Fast SYBR Green Master Mix (Thermo Fisher Scientific) on the StepOnePlus Real-Time PCR System v2.2 (ABI, Thermo Fisher Scientific). The expression levels of tested genes were normalized to previously validated housekeeping gene (Columbus-Shenkar et al. 2018), and the relative gene expression was calculated using the 2ΔΔCt method. The significance level was calculated by two-tailed Student's *t*-test to ΔCt values for each of the pairwise comparisons to control shRNA.

### Assessment of Phenotype Following KD of MACs

We injected either a specific shRNA or a combination of shRNAs. Control shRNA were injected in parallel at an equal concentration, and morphology was tracked for injected animals until 10 dpf. Each experiment consisted of at least three biological replicates. For the combinations of shRNAs to target MAC1, 3, and 4, 400 ng/µL was injected of each specific shRNA, whereas 1200 ng/µL of control shRNA was injected in parallel. After 10 dpf, animal physiology was visualized under an SMZ18 stereomicroscope equipped with a DS-Qi2 camera (Nikon, Tokyo, Japan).

### ISH

ISH was performed as previously described (Genikhovich and Technau 2009b). Embryos older than 4 d were treated with 2 u/µL T1 RNAse (Thermo Fisher Scientific) after probe washing in order to reduce background. Stained embryos and larvae were visualized with an Eclipse Ni-U microscope equipped with a DS-Ri2 camera and an Elements BR software (Nikon). For each gene, at least 20 individuals from each developmental stage were tested. The specificity of the NveMAC1 probe was confirmed by performing ISH on 4 dpf animals that were injected with either control shRNA or shRNA to knockdown MAC1. This was repeated and the ratio of stained animals was compared.

### Meta-analysis of Bulk RNA-seq scRNA-across

We performed a comparative analysis using previously published RNA-seq data of two reporter lines and three mutant lines. Data from both reporter lines were generated from *Nematostella* primary polyps that express a fluorescent transgene, under either the promoter of NvNcol3::memOrange2, a nematocyte maker (Sunagar et al. 2018; Gahan et al. 2022), or NvElav1::memOrange, a neuronal marker (Nakanishi et al. 2012; Tournière et al. 2020). The data from mutant lines consisted of HOX homozygote knockout lines (ANthox1a, ANthox6a, ANthox8) which play an important role in regulating endomesodermal segmentation in *Nematostella* (He et al. 2023). Raw reads were downloaded from the SRA (NvNcol3::memOrange2: PRJEB40304, NvElav1::memOrange PRJEB36771, HOX mutants: PRJNA727015). Raw reads were trimmed and quality filtered by Trimmomatic. Reads were mapped to a modified *Nematostella* genome. Mapping was performed using STAR and the gene counts quantified using RSEM. Differential expression analyses were performed using scripts from Trinity using both DESeq v2.139 and edgeR v3.1675. Gene models used in all downstream analyses were from previously published annotations (Schwaiger et al. 2014). Differentially expressed genes were defined by false discovery rate (FDR) < 0.05 and fold change ≥ 2. Genes identified by both methods were considered as differentially expressed. Biological replicates were quality-checked for batch effect using sample correlation and PCA.

### Structural Predictions

AlphaFold2 was used to model the structure of *P. semoni* MACs and NveMACs 1 to 7 (Jumper et al. 2021). Top-ranked AlphaFold2 models for each MAC were used as a query to search the AlphaFold/UniProt50 and Protein Data Bank (PDB) database using the Foldseek webserver in TM-align mode (van Kempen et al. 2023). Predicted structure figures were generated using PyMOL version 2.4.0 (Schrödinger, LLC).

## Supplementary Material

msae082_Supplementary_Data

## Data Availability

The data used in this article is available in its online Supplementary material.
