# Supplementary material for: Sea Anemone Membrane Attack Complex/Perforin Superfamily Demonstrates an Evolutionary Transitional State between Venomous and Developmental Functions
Source: Mol Biol Evol. 2024 Apr 27;41(5):msae082. doi: 10.1093/molbev/msae082 (PMC11090067; doi:10.1093/molbev/msae082)
Supplement: msae082_Supplementary_Data [file msae082_supplementary_data.zip › Supplementary Figures Surm et al.pdf]

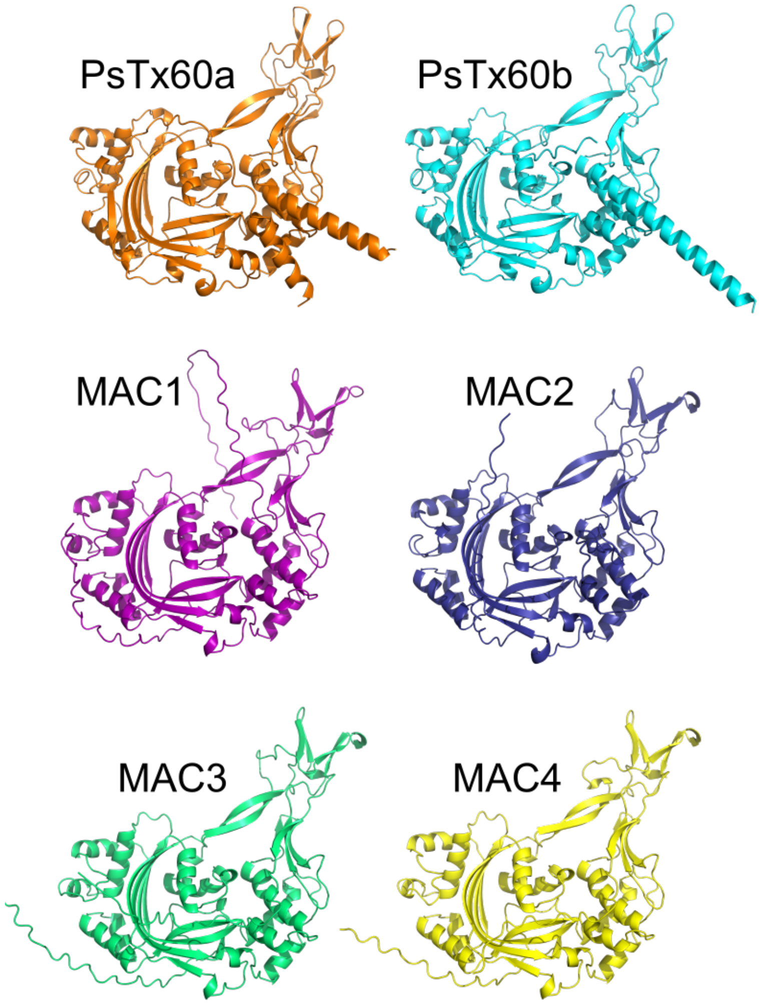

Fig. S1. Alphafold Predicted structures of MACPF-encoding proteins in *Phyllodiscus semoni* (PsTx60a and PsTx60b) and *Nematostella vectensis* (MAC1-4).

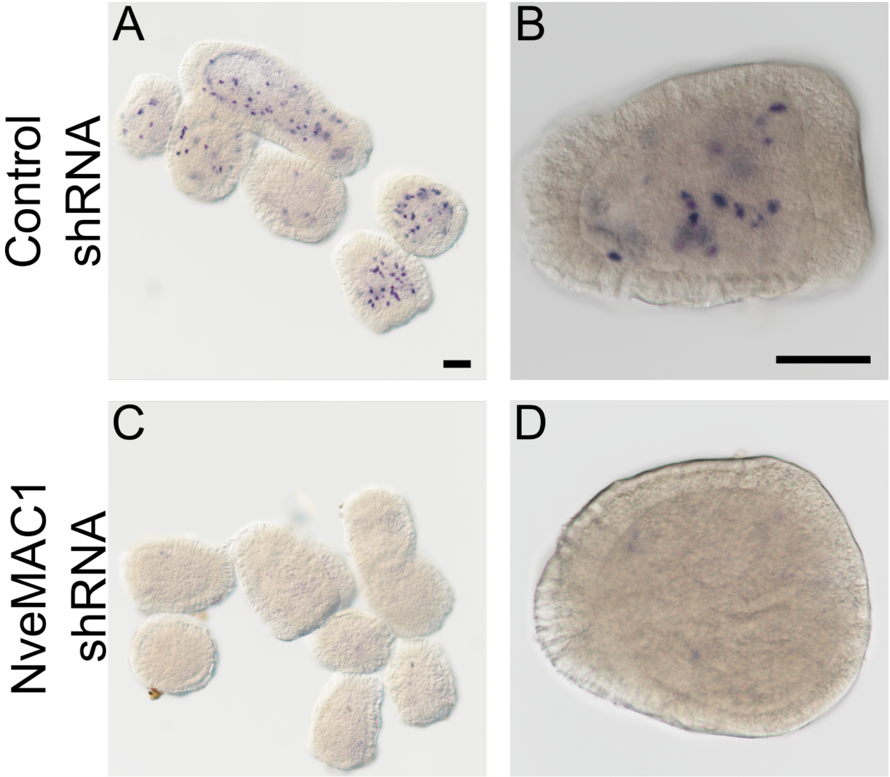

Fig. S2. In situ hybridization probe specificity validation. Multiple (A) and an individual (B) 4dpf *Nematostella* stained using MAC1 probe following injection of control shRNA. Multiple (C) and an individual (D) 4dpf *Nematostella* stained using MAC1 probe following injection of shRNA targeting NveMAC1. Scale bars, 100 µm.

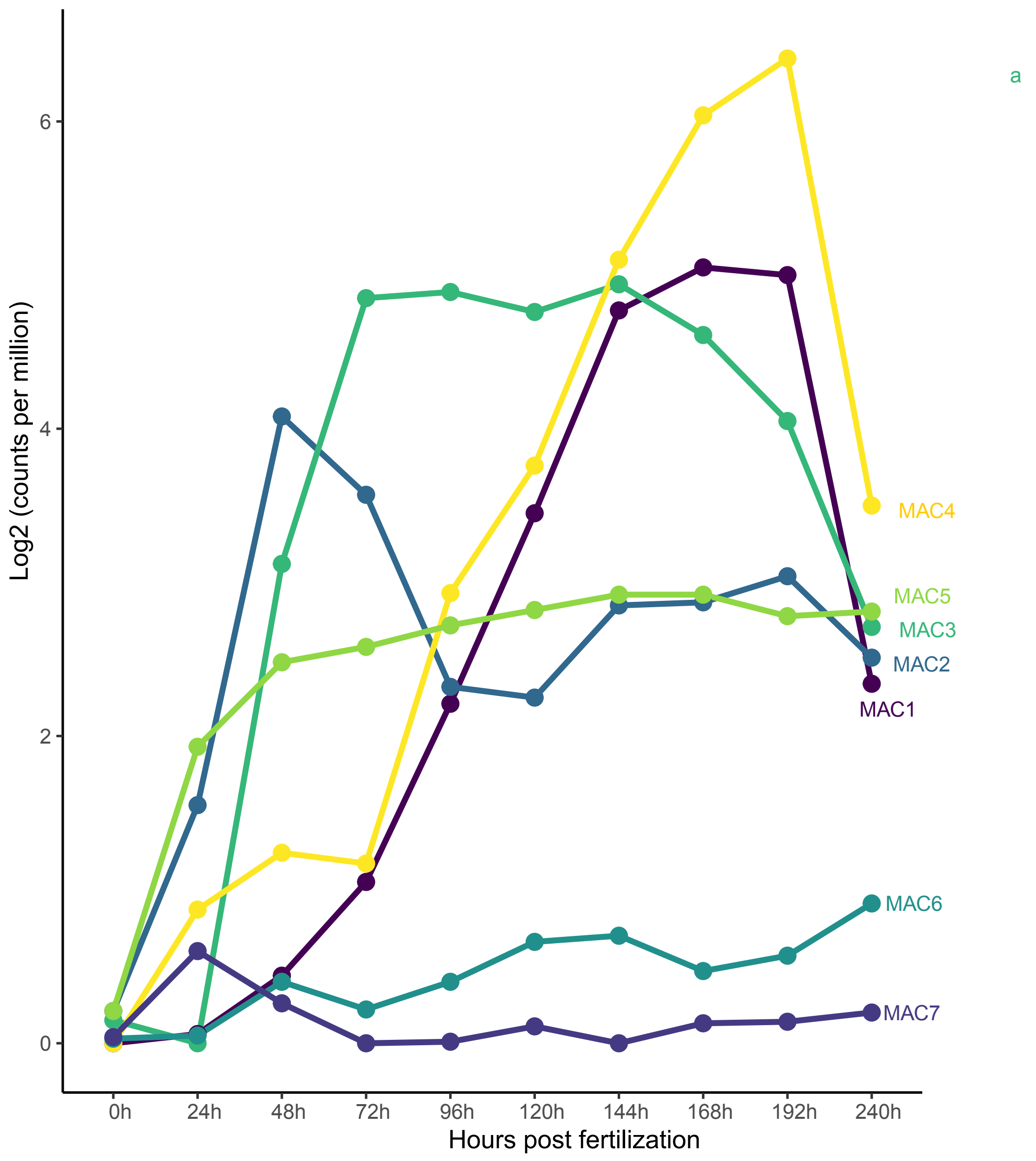

Fig. S3 Graph of RNA levels of seven MACPF genes throughout embryonic development of Nematostella taken from the NvERTx database

Table S1.

Meta analysis and scRNA-seq and bulk RNA-seq across Anthozoa

Table S2.

Genomic organization of MACPFs in from Anthozoan genomes and macrosynteny with *Nematostella* and *Scolanthus* chromosomes.

Table S3.

Endo-atlas expression of *Nematostella* toxins and MACPFs.

TableS4.

nCounter gene expression of NveMACs and housekeeping genes (HKG) across development for both raw (A) and normalized (B).

TableS5.

RNA-seq gene expression of NveMACs across development as hours post fertilization (hpf) generated from NvERTX

TableS6.

Knockdown efficiency of shRNA and their NveMAC target as well as combined knockdown of NveMAC1, 3 and 4.

TableS7.

Settlement ratio of 10dpf *Nematostella* polyps using a shRNA targeting NveMACs 1-4.

Table S8.

Downregulation of NveMACs generated using RNA-seq following knockout of HOX genes in *Nematostella* that regulate endomesodermal segmentation.
